# The spatiotemporal pattern of earthworm community in the grass savannas of Lamto (Ivory Coast)

**DOI:** 10.3897/BDJ.3.e6515

**Published:** 2015-11-13

**Authors:** Jean-Pierre Rossi, Patrick Lavelle

**Affiliations:** ‡ INRA, UMR CBGP, Montferrier-sur-Lez, France; § Université Pierre et Marie Curie (Paris 6), Paris, France

**Keywords:** Earthworm community, space-time data, multivariate analysis, grass savanna, Eudrilidae, *Millsonia anomala*, *Dichogaster agilis*, west Africa

## Abstract

**Background:**

The impact of earthworms on both soil physical properties and soil organic matter dynamics has been well documented (Lavelle and Spain 2001). There is a wealth of literature dedicated to the biological mechanisms at work or to empirical approaches based on field data. Assessing the functional role of a species or community implies establishing both time and space scales at which it is effectively the primary determinant of the process(es) at hand. In that context, space-time data analyses are powerful tools to process community data collected on numerous occasions but are, however, not widely disseminated in the community of ecologists. Although computer resources are available, one difficulty is that *ad hoc* field data are not always easily available which hinders the percolation of the methods.

**New information:**

We provide the results of a 5 dates survey of earthworm community in a grass savanna of Lamto (Ivory Coast) conducted between 1995 and 1997. At each sampling date, earthworm community was assessed by hand-sorting a set of 100 soil monoliths distributed on a regular grid of 5 m mesh. These data were analyzed in Rossi (2003a) and are published here with the aim that they could be reanalyzed using new statistical tools (e.g. MEM analyses see Jiménez et al. 2014) or serve as example for researchers that train on space-time statistical methods.

## Introduction

Earthworms play an important role is soil functioning because they affect soil physical structure, soil matter dynamics and interact with other soil dwellers (Lavelle and Spain 2001). Assessing their functional role implies establishing both time and space scales at which they constitute the primary determinant of soil processes. A common strategy to tackle this question is to adopt a two steps approach involving a global spatiotemporal analysis of the community followed by an analysis of the co-structures shared by the community a the set of environmental variables conveying the processes at hand (Rossi 2003a).

It is beyond the scope of the present paper to discuss the statistical approaches that allow to perform such data processing. Various tools are available and the Partial Triadic Analysis (PTA) has proved to be effective on several occasions (Rossi 2003a, Jiménez et al. 2006). It provides useful summaries of the space-time community structure that can be compared to soil environmental descriptors. In a nutshell, the PTA searches for the community spatial structure common to all sampling dates (referred to the “compromise” in the jargon of the PTA). A complementary step (referred to as the intra-structure analysis) examines the discrepancies between the observed pattern recorded at each sampling occasions and the model common to all dates. Readers are referred to Rossi (2003a) and Rossi et al. (2014) for a presentation of the PTA.

Because space-time analyses are still not familiar to many biologists, we believe that an educational effort is welcome. One reason why the dissemination of such methods is still limited while computer resources are available (Chessel et al. 2004) is that *ad hoc* field data are not always easily available. The purpose of the present paper is to fill this gap by providing the original raw data set used in Rossi (2003a). This will facilitate the percolation of space-time multivariate methods in community ecology and help biologist who learn the PTA and related methods. In addition, we hope that making our raw data widely available will allow re-analyses by means of new tools and give rise to meaningful discussions (e.g. MEM analysis, see Jiménez et al. (2014)). Note also that part of the present data set was used to illustrate the SADIE method in Rossi (2003b).

## Sampling methods

### Study extent

**Figure 1.**
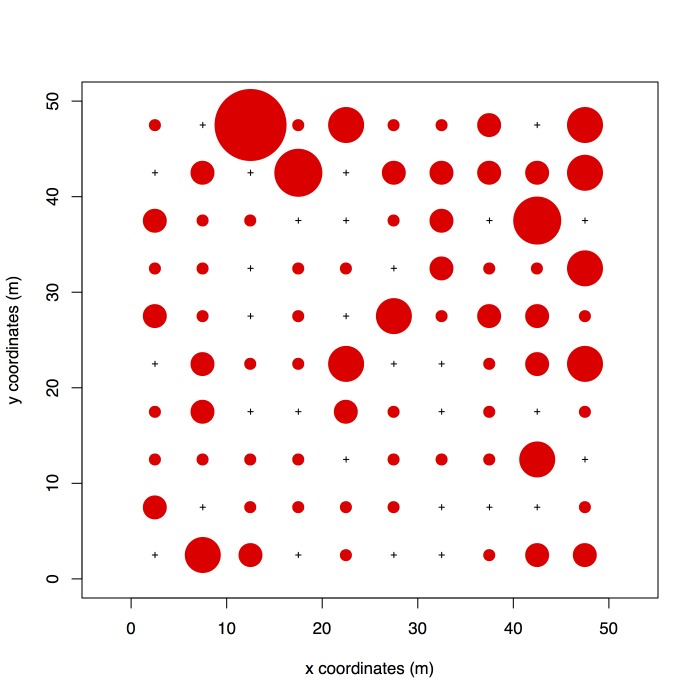
Spatial distribution of the endogeic earthworm *Millsonia anomala* in the upper 10 cm of soil a grass savanna. Sampling was carried out in May 1995 in Lamto (Ivory Coast). The data are available given in Suppl. material 1​. The density ranges from 0 (crosses) to 96 (largest red circle) individuals per m^2^.

The data were collected a grass savanna (*Loudetia simplex*) in the Station d’Ecologie Tropicale de Lamto (Ivory Coast) (5°02’W, 6°13’N) at a place known as “le virage glissant”. The mean annual rainfall is ca 1200 mm and the mean temperature 28 °C. A dry season occurs from December to February and a rainy season from March to November interrupted by a decrease in rainfall during August. We sampled earthworm community within a plot of 45 × 45 m which was randomly located within a large area covered with *L. simplex* and sparse palm trees (*Borassus aethiopium*) (Fig. 1). The plot was sampled on 5 occasions: May and November 1995, June and December 1996 and June 1997. Each sampling campaign was carried out during the rainy season when earthworm populations have reached their highest density and biomass in Lamto (Lavelle 1978).

### Sampling description

Earthworms were sampled by hand-sorting a 25 × 25 × 10 cm soil monolith. Hand-sorting was carried out in the field where specimens were identified, counted and released in the soil. Samples were taken in a 10 × 10 grid with a mesh size of 5 m. Since earthworm sampling was carried out on 5 dates, samples were displaced, from one date to another, along a spiral whose origin was represented by the point sampled at the first date. In so doing we avoided taking two soil monoliths exactly at the same location (Rossi 2003a​). The sample coordinates were considered as identical from one date to another (Suppl. material 1). Distinguishing *C. zielae* and *S. porifera* requires adult individuals with visible external sexual organs. Because these conditions were not always met and because we performed rapid identification in the field, both species were recorded as a single taxa referred to as the Eudrilidae group. Earthworms (except eudrilidae) were distributed among broad age class categories: adults, sub-adults and juveniles (Suppl. material 1).

## Geographic coverage

### Description

The grass savanna in the Station d’Ecologie Tropicale de Lamto (Ivory Coast) (5°02’W, 6°13’N).

## Taxonomic coverage

### Description

The earthworm community of the grass savannas in Lamto comprises several species (Lavelle 1978) amongst which the most frequent are two species of the Eudrilidae family (*Chuniodrilus zielae* (Omodeo) and *Stuhlmannia porifera* (Omodeo & Vaillaud)), the megascolecid *Millsonia anomala* (Omodeo) the dominant species in terms of biomass (Fig. 1) and the epigeic megascolecid *Dichogaster agilis* (Omodeo & Vaillaud). Readers are referred to Lavelle (1978) for more details about the ecology of earthworm species in Lamto.

## Temporal coverage

### Notes

sampling was carried out on 5 occasions: May and November 1995, June and December 1996 and June 1997.

## Usage rights

### Use license

Creative Commons CCZero

## Data resources

### Data package title

Earthworm community in the grass savanna of Lamto (Ivory Coast)

### Number of data sets

1

### Data set 1.

#### Data set name

Earthworm density

#### Number of columns

23

#### Description

**Data set 1.** 

Column label	Column description
sampling point	sampling point number
x coordinates (m)	the x coordinates of sampling locations
y coordinates (m)	the y coordinates of sampling locations
Eudrilidae	counts: individuals per sampling unit for the Eudrilidae
Millsonia anomala (adults)	counts: individuals per sampling unit for Millsonia anomala (adults)
Millsonia anomala (subadults)	counts: individuals per sampling unit for Millsonia anomala (subadults)
Millsonia anomala (juveniles)	counts: individuals per sampling unit for Millsonia anomala (juveniles)
Millsonia anomala (cocoons)	counts: individuals per sampling unit for Millsonia anomala (cocoons)
Dichogaster agilis (adults)	counts: individuals per sampling unit for Dichogaster agilis (adults)
Dichogaster agilis (subadults)	counts: individuals per sampling unit for Dichogaster agilis (subadults)
Dichogaster agilis (juveniles)	counts: individuals per sampling unit for Dichogaster agilis (juveniles)
*Agastrodrilus* sp.	counts: individuals per sampling unit for *Agastrodrilus* sp.
other	counts: individuals per sampling unit for other earthworms
Eudrilidae	abundance: individuals per m2 for the Eudrilidae
Millsonia anomala (adults)	abundance: individuals per m2 for Millsonia anomala (adults)
Millsonia anomala (subadults)	abundance: individuals per m2 for Millsonia anomala (subadults)
Millsonia anomala (juveniles)	abundance: individuals per m2 for Millsonia anomala (juveniles)
Millsonia anomala (cocoons)	abundance: individuals per m2 for Millsonia anomala (cocoons)
Dichogaster agilis (adults)	abundance: individuals per m2 for Dichogaster agilis (adults)
Dichogaster agilis (subadults)	abundance: individuals per m2 for Dichogaster agilis (subadults)
Dichogaster agilis (juveniles)	abundance: individuals per m2 for Dichogaster agilis (juveniles)
*Agastrodrilus* sp.	abundance: individuals per m2 for *Agastrodrilus* sp.
other	abundance: individuals per m2 for other earthworms

## Supplementary Material

Supplementary material 1 Earthworm community in upper soil of a grass savanna in Lamto (Ivory Coast) Data type: Space-time density data
Brief description: The data set provides both count data and the corresponding abundances (individuals per m^2^) for the earthworms sampled in the upper soil (10 cm depth) in a grass savana (Lamto, Côte d'Ivoire). Sampling was carried out on 100 sampling points located on a square grid with spacing of 5 m. The grid was sampled on 5 occasions: May and November 1995, June and December 1996 and June 1997. Sampling coordinates are given in meters.
File: oo_55683.xls J.-P. Rossi and P. Lavelle
